# Hydrogen Sulfide-Releasing Fibrous Membranes: Potential Patches for Stimulating Human Stem Cells Proliferation and Viability under Oxidative Stress

**DOI:** 10.3390/ijms19082368

**Published:** 2018-08-11

**Authors:** Ilaria Cacciotti, Matteo Ciocci, Emilia Di Giovanni, Francesca Nanni, Sonia Melino

**Affiliations:** 1 Department of Engineering, University of Rome “Niccolò Cusano”, via Don Carlo Gnocchi 3, 00166 Rome, Italy; 2 Italian Interuniversity Consortium on Materials Science and Technology (INSTM), 50121 Florence, Italy; 3 CIMER Center for Regenerative Medicine, University of Rome Tor Vergata, via Montpellier 1, 00133 Rome, Italy; 4 Department of Chemical Science and Technologies, University of Rome “Tor Vergata”, via della Ricerca Scientifica1, 00133 Rome, Italy; ciocci.matteo@gmail.com (M.C.); emilia.digiovanni8@gmail.com (E.D.G.); 5 Enterprise Engineering Department, University of Rome “Tor Vergata”, via del Politecnico 1, 00133 Rome, Italy; fnanni@ing.uniroma2.it

**Keywords:** PLA fibers, organosulfur compounds, garlic extracts, mesenchymal stem cells, microstructure, thermal and mechanical properties, cytotoxicity, antibacterial properties

## Abstract

The design of biomaterial platforms able to release bioactive molecules is mandatory in tissue repair and regenerative medicine. In this context, electrospinning is a user-friendly, versatile and low-cost technique, able to process different kinds of materials in micro- and nano-fibers with a large surface area-to-volume ratio for an optimal release of gaseous signaling molecules. Recently, the antioxidant and anti-inflammatory properties of the endogenous *gasotramsmitter* hydrogen sulfide (H_2_S), as well as its ability to stimulate relevant biochemical processes on the growth of mesenchymal stem cells (MSC), have been investigated. Therefore, in this work, new poly(lactic) acid fibrous membranes (PFM), doped and functionalized with H_2_S slow-releasing donors extracted from garlic, were synthetized. These innovative H_2_S-releasing mats were characterized for their morphological, thermal, mechanical, and biological properties. Their antimicrobial activity and effects on the in vitro human cardiac MSC growth, either in the presence or in the absence of oxidative stress, were here assessed. On the basis of the results here presented, these new H_2_S-releasing PFM could represent promising and low-cost scaffolds or patches for biomedical applications in tissue repair.

## 1. Introduction

One of the main targets of regenerative medicine is the emulation of the physiological environment by fine-tuning of an array of biochemical and physical stimuli in order to improve stem cells proliferation and differentiation. Many factors with the ability to affect the biological mechanisms of stem cells have already been identified, due to their potential relevance in contributing to generate the optimal array of stimuli for driving the cell fate. Nevertheless, the differentiating potential of many other factors remains neglected. In particular, the effects of gaseous signaling molecules, such as hydrogen sulfide (H_2_S), on adult stem cells remain to be investigated. Hydrogen sulfide is an endogenously produced biological agent belonging to the *gasotransmitters* family. H_2_S plays pivotal roles in the central nervous, respiratory, and cardiovascular systems, where it exerts relevant protective effects. In the last decade, it has been demonstrated that H_2_S is a physiological mediator able to limit inflammation and free radical damage [1] by reacting with multiple oxidant stressors including: peroxynitrite [1], superoxide radical anion [2], and hydrogen peroxide [3]. H_2_S is able to activate the Nuclear factor (erythroid-derived 2)-like 2 (Nrf2)-antioxidant response element (ARE) pathway that is associated to an adaptive response of the cell against oxidative stress [4]. Moreover, this signaling molecule is also able to produce glutathione persulfide (GSSH) in mitochondria [5,6,7], a more efficient H_2_O_2_ scavenging molecule than glutathione (GSH). Therefore, H_2_S-donors have acquired a great therapeutic potential for widely diffused pathologies, such as neurodegenerative [8,9,10], cardiovascular [11,12,13], and gastrointestinal diseases [14,15].

Endogenously, H_2_S is produced in mammalian tissues from l-cysteine or polysulfides metabolism [16]. In particular, the metabolism of organosulfur compounds (OSCs) derived from garlic leads to the production of H_2_S slow-releasing donors with antimicrobial, antioxidant [17,18] and anti-inflammatory properties [19], heart protection, chemo-sensitization features and in vitro inhibition of tumour cells proliferation through the induction of apoptosis [19]. Moreover, their H_2_S-release can also be prolonged and potentiated by biological thiols that are normally present in the biological systems such as: protein-thiols groups, cysteine, and glutathione. On this basis, the garlic OSCs can be considered promising biomolecules to provide antimicrobial/antioxidant properties to scaffolding materials able to support the stem cell differentiation, improving their biological responsiveness in tissue repair and regeneration.

The fabrication of bioactive materials able to fulfil a double function, playing both support and stimulation of the biochemical processes by releasing of signaling molecules, represents a novel approach in the scaffolds production. H_2_S-releasing materials could exert a wide range of protective actions, vasodilation, angiogenesis, antioxidant, and anti-inflammatory effects, improving the regenerative capacity of tissue engineering grafts, especially in cardiovascular systems [20,21,22,23]. Recently, it was demonstrated that H_2_S-functionalized hydrogels and fibers are able to enhance the proliferation of human cardiac progenitor/stem cells [24], cardiomyocytes and immortalized fibroblast cell lines [25], and to improve the wound healing process [26].

Fibrous structures are able to mimic the tissue extracellular matrix (ECM) morphology and are very suitable to entrap and stabilize thermo-labile substances, occurring at room temperature and ensuring their controlled release. Nanofibers can be easily obtained by electrospinning technique, which is a low-cost, user friendly, and versatile process. Moreover, it allows to process different kinds of materials in fibers, obtaining fibrous structures with diameters ranging from nanometers to microns and large surface area-to-volume ratio [19,27,28,29]. Among the different biopolymers, poly(lactic) acid (PLA) is widely used, since it is a FDA (Food and Drug Administration) approved biocompatible and biodegradable polymer with a high hydrophobicity and linear structure that confer excellent spinnability for fabrication of fibrous biomats by electrospinning technique.

Therefore, in this work, two different approaches were used in order to generate electrospun PLA fibrous membranes (PFM) able to provide H_2_S slow-release: (i) the direct doping of PFM with natural garlic OSCs by drop casting garlic oil-soluble extract (GaOS) on the surface of neat fibrous mats and (ii) the optimization of a protocol for the “*ex novo*” production of OSCs-functionalized fibers, by OSCs direct encapsulation within PFM, using both GaOS and diallyl disulfide (DADS). GaOS doped PFMs were here characterized and analyzed for their H_2_S-release ability and for their effect on in vitro cell proliferation of human Lin^−^ Sca1^+^ cardiac mesenchymal stem cells (cMSC). Moreover, PFM functionalized with GaOS and DADS were produced using a novel protocol for their synthesis and characterized for their morphological, thermal, mechanical, and biological properties. The data here presented show that the produced H_2_S-releasing fibers can be useful for the optimization of nano-structured patches for tissue repair.

## 2. Results and Discussion

### 2.1. GaOS Extract Production and Characterization

**Figure 1:**
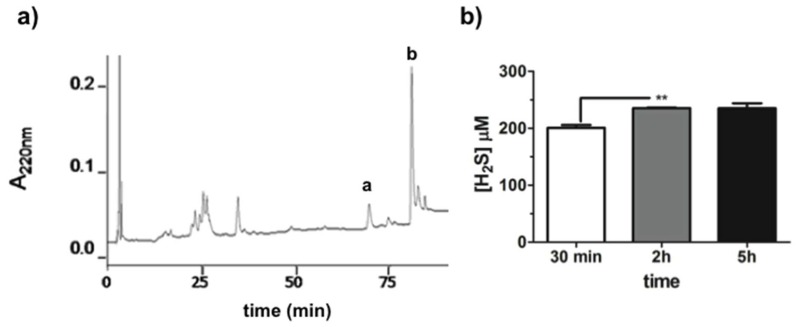
Characterization of the GaOS extract. (**a**) RP-HPLC chromatogram of the GaOS obtained using C_18_ column at 0.8 mL/min flow rate. The elution was performed with a linear gradient of solv. B (80% CH_3_CN, 0.1% TFA). Peaks a and b are characteristic of DADS; (**b**) H_2_S-release by 25 μL of GaOS after 30 min, 2 h and 5 h of incubation at 37 °C in the presence of 1 mM DTT and detected by MB assay. ** p < 0.02.

GaOS was obtained using a previously optimized extraction protocol [30]. It was characterized by the first step of extraction of the water-soluble fraction at low temperature in order to reduce oxidative reactions. The obtained ethanol-soluble fraction (GaOS) was filtered, analyzed by RP-HPLC (Figure 1a) and stored at −20 °C. About 2 mL of GaOS solution, with a concentration of 42 mg of dried weight (d.w.)/mL, were obtained for each extraction from 5 g of garlic. The RP-HPLC analysis of the GaOS, shown in Figure 1a, indicated the presence of a main hydrophobic component (corresponding to the peaks a and b), eluted at the same retention time of DADS (see Figure S2), which represents the major constituent (about 60%) of the oil-garlic fraction [31]. Garlic OSCs and their conjugates have been studied as optimal H_2_S slow-releasing donors [30,32,33,34]. Therefore, the ability of the GaOS solution to release H_2_S was here assessed by Methylen Blue (MB) assay after 30 min, 2 h and 5 h of incubation at 37 °C (Figure 1b).

In particular, the H_2_S was released in a time dependent manner, and 25 μL of GaOS led to a production of 200.8 ± 8.3 μM (±SD) of H_2_S after 30 min of incubation at 37 °C. This result was in agreement with the already studied property of the garlic OSCs to generate H_2_S with a slow-releasing rate [33]. The maximum of the H_2_S-release was obtainable after 2 h of incubation, as shown in Figure 1b, since there was not an increase of the H_2_S-production after 5 h of incubation. Therefore, for this property and its easy production, GaOS could be of potential interest for the production of biocompatible systems for the H_2_S controlled release in therapeutic applications. Indeed, although the pharmacological properties of this signaling molecule have been established, its administration is not easy and is greatly limited by the difficulty of ensuring an accurate posology control and the risk of overdose. On this context, here we fabricated biocompatible and biodegradable PFM able to embed GaOS and allow a more controlled H_2_S slow-release.

### 2.2. Synthesis of GaOS Doped PFM as H_2_S-Releasing and Antimicrobial Fibrous-Mats

In the last years, there has been a growing interest towards plant oil-extracts with biological activity and the production of related delivery systems for several applications [35,36,37,38,39,40,41,42,43,44,45]. Among them, electrospun fibrous mats have attracted a lot of attention, being ideal for trapping and stabilizing bioactive molecules at room temperature, providing a slow-release of organic molecules and volatile compounds.

**Figure 2:**
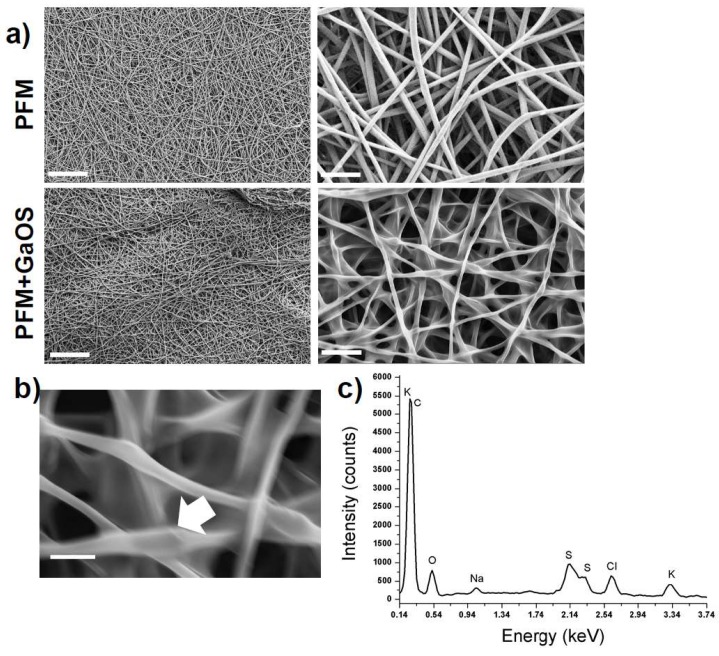
Microstructural characterization of the neat and GaOS doped PLA fibrous membranes (PFM). (**a**) SEM micrographs of PFM and PFM+GaOS (**left**: magnification 1k×, scale bar 50 µm, **right**: magnification 10k×, scale bar 5 µm); (**b**) SEM micrograph (magnification 30k×, scale bar 2 µm) and (**c**) energy dispersive X-ray (EDS) spectrum of PFM+GaOS. The white arrow indicates the area submitted to the EDS microanalysis.

In Figure 2a the scanning electron microscopy (SEM) micrographs of the neat PFM are shown, evidencing the presence of defect-free randomly oriented fibers with an average diameter of 0.71 ± 0.18 µm (Table 1).

**Figure 3:**
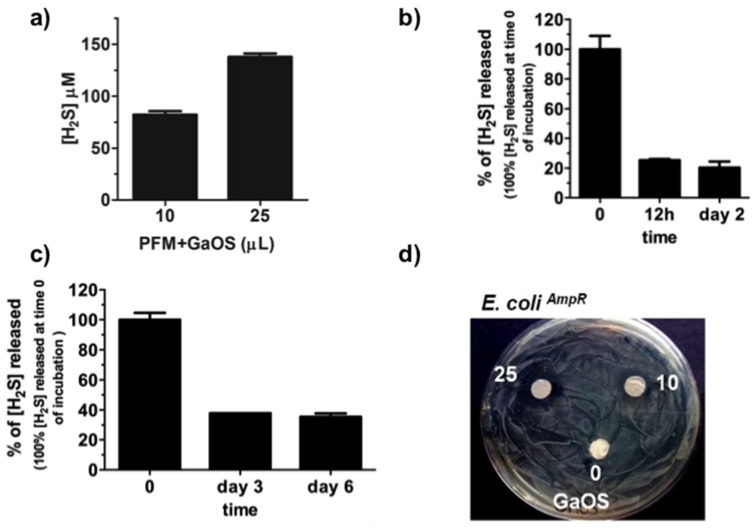
H_2_S slow-release and antimicrobial activity of PFM+GaOS. (**a**) H_2_S-release from PFM doped with 10 and 25 μL of GaOS and dried; the values of PFM alone were subtracted. H_2_S-release over time from PFM+GaOS, doped with 25 μL of GaOS and dried, after incubation: (**b**) in 50 mM Tris-HCl buffer, pH 8.0 for 0, 12 h, 2 days; (**c**) dried in a petri dish at 25 °C for 0, 3 and 6 days; (**d**) Photo-optical image of the *E. coli^AmpR^* growth in agar-LB medium in the presence of PFM disks (1 cm diameter) doped with 0, 10, 25 μL of GaOS (42 mg/mL).

The PFM were doped with 25 μL of GaOS (42 mg/mL) and dried (PFM+GaOS). The GaOS addition slightly modified the fiber morphology, leading to thinner fibers and, consequently, higher fibers packing density (Figure 2a,b). Furthermore, the energy dispersive X-ray (EDS) spectrum of the PFM+GaOS, acquired after about 3 weeks from the doping and without special storage, demonstrated the presence of OSCs embedded within the PFM porous network, via detection of sulfur (Figure 2c) that was not present in the EDS spectrum of PFM without GaOS (Figure S3). These results confirmed a great compatibility between the PFM and the OSCs, guaranteeing a good OSCs adhesion and entrapment within the fibers. Therefore, the H_2_S release from the GaOS conditioned PFM was investigated by MB assay. The doped PFM were able to release H_2_S in a concentration dependent manner, as shown in Figure 3a.

The production of H_2_S was 137.8 ± 3.3 (±SD) µM after 1 h shaking at 37 °C, which was little less than that obtained from the GaOS solution after 30 min, even demonstrating that the PFM got a high ability to embed the GaOS extract. The H_2_S release from the PFM+GaOS was also performed over time both after incubation at 37 °C in the buffer for 0, 12 h, and 2 days, and after 0, 3, and 7 days putting them in a petri dish at room temperature. After 2 days of incubation in buffer the membranes were able to release the 20.4% of the H_2_S, while after 6 days the dried PFM+GaOS were able to release the 35.4% of the H_2_S produced immediately after doping. These results are in agreement with the demonstrated trapping ability of fibrous membranes, and demonstrate that the H_2_S slow-release can be improved embedding the GaOS within the PFM.

Furthermore, since the demonstrated antimicrobial properties of the garlic extracts could represent a key feature for preventing microorganism colonisation and biofilm formation [46], the antibacterial behaviour of the PFM+GaOS was here investigated. The doped PFM were able to inhibit the proliferation of the BL21 *E. coli^AmpR^* strain in a concentration dependent manner, as it was observable from the absence of biofilm around the GaOS doped PFM disk (Figure 3d).

Therefore, H_2_S-releasing mats with antimicrobial properties, such as GaOS doped PFM, could disclose attractive pharmacological perspectives also in tissue repair and regeneration. In general, the effects of H_2_S-donors on stem cells have not been widely investigated yet, and even more the effects of H_2_S slow-releasing biomats [24,25,26,47]. In this context, the influence of the H_2_S-releasing PFM on adult stem cells was here assessed.

### 2.3. GaOS Doped PFM Improve the cMSC Proliferation

**Figure 4:**
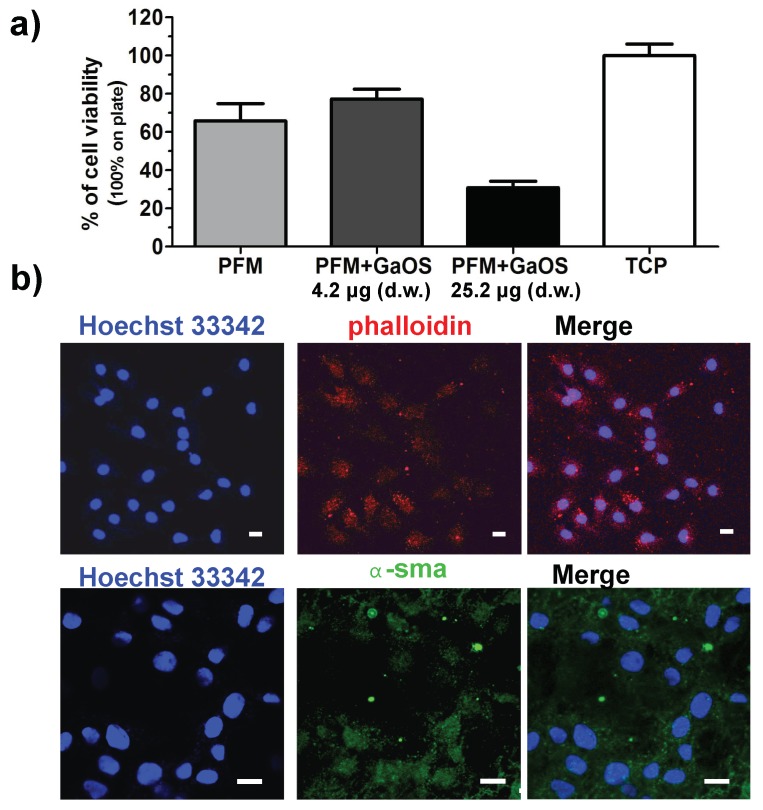
PFM+GaOS as scaffolds for cMSC cultures. (**a**) Cell viability of cMSC seeded on PFM disks (0.5 cm of diameter) with 0 μg, 4.2 μg (d.w.) and 25.2 μg (d.w.) of GaOS after 3 days of growth; (**b**) fluorescence confocal micrographs of cMSC cultured on PFM+GaOS with 4.2 μg (d.w.) of GaOS for 3 days. The nuclei are stained with Hoeschst 33342 (in blue) and the expressions of α-sma (in green) and phalloidin (in red) proteins are detected. Scale bars = 10 μm.

The PFM+GaOS disks were employed as scaffolds for 2D cultures of cMSC in order to investigate their effect on the stem cell viability and proliferation. After 3 days of growth, the cell viability of cMSC seeded onto the PFM+GaOS with low concentration of GaOS (4.2 μg d.w.) was increased with respect to the cells seeded on neat PFM (Figure 4a).

The results were expressed as a percentage of the control, represented by cells growth on a tissue culture plate (TCP). Figure 4b shows the fluorescence micrographs of cMSC cultured on PFM+GaOS (4.2 μg of d.w.) after 3 days of growth; the nuclei and the cytoskeleton were stained with Hoechst and phalloidin, respectively. The cells were dispersed on the membrane and a significant production of α-smooth muscle actin (α-sma) was observed, indicating a favourable interaction between the cells and the functionalized biomaterial (cMSC on PFM are shown in Figure S4). By contrast, a cytotoxic effect was revealed using PFM doped with a higher concentration of GaOS (25.2 μg of d.w.). These results are in agreement with the effects of the H_2_S on the cell growth described in the literature [25]. Indeed, although the exogenous administration of H_2_S elicits a wide range of protective effects including anti-inflammatory, antioxidant, and down regulation of under stress cellular metabolism, [20,23] by contrast the direct contact with high levels of exogenous H_2_S can induce cytotoxicity [25,26].

**Figure 5:**
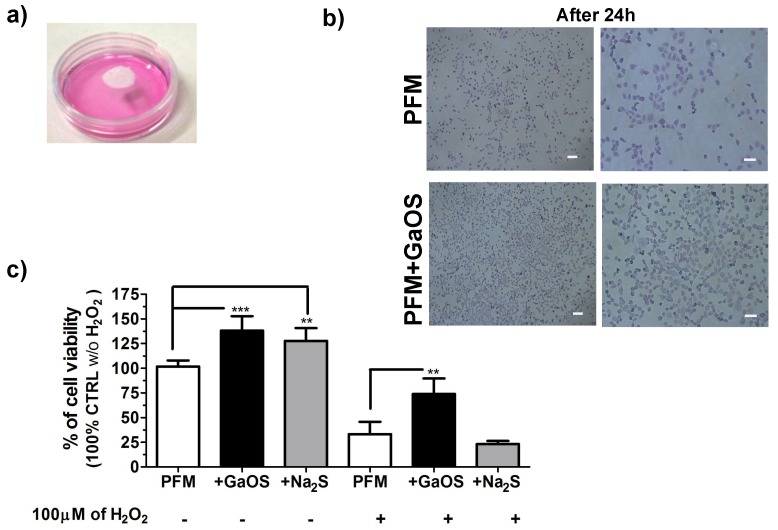
Effect of gaseous release from PFM+GaOS on 2D-culture of cMSC. (**a**) Photo-optical image of PFM+GaOS on the lids of the petri dishes where 1 × 10^4^ cells/cm^2^ were seeded; (**b**) pptical micrographs of cMSC after 24 h of cell growth in the presence of PFM+GaOS disk (1 cm of diameter) on the petri dishes-lid and stained with crystal violet; (**c**) cell viability of cMSC cultures in the presence or in the absence of 100 µM H_2_O_2_ with PFM, PFM+GaOS, or PFM + Na_2_S on the petri dishes-lids. ** p < 0.02; *** p < 0.005.

In order to better clarify the effects of the PFM+GaOS and their H_2_S-releasing ability on cell proliferation of cMSC, other experiments were performed without a direct contact of the PFM with the cells. PFM, with and without 25 μL of a diluted GaOS solution (19.8 mg of d.w./mL) releasing 95 μM of H_2_S, were placed to the centre of the lids of petri dishes where 10,000 cells/cm^2^ were seeded, as shown in the photo-optical image in Figure 5a. After 24 h of cell growth, a high increase of the proliferation in the presence of PFM+GaOS was observed, as shown in Figure 5b, where the cMSC were fixed and stained with crystal violet. Accordingly, a statistically significant increase of the cell viability was also observed in the presence of PFM+GaOS by means of MTT assay (Figure 5c). In order to compare the effect of PFM+GaOS with the PFM embedding a pure and fast H_2_S-releasing agent, the cell growth was assessed in the presence of PFM doped with 25 μL of 95 μM Na_2_S. Na_2_S was immediately released from PFM as H_2_S. Also in this last case a significant increase of the cell growth with respect to the neat PFM was observed in the absence of H_2_O_2_ (Figure 5c).

These results indicated that H_2_S-releasing PFM+GaOS stimulated the cMSC proliferation through the release of gas molecules and without a direct contact with the cells.

The more stimulating effect of PFM+GaOS on cell growth with respect to the PFM + Na_2_S could be related to several factors, such as: (i) a slower H_2_S-production from the sulfane sulfur compounds present in the garlic extract, that is dependent from hydrolysis or nucleophilic substitution [33]; (ii) a better trapping into the fibres of GaOS, due to its hydrophobicity; and (iii) possible additional H_2_S independent pathways.

Moreover, taking into account the ability of H_2_S-releasing donors to reduce the cellular oxidative damages [48], the effect of PFM+GaOS on cell growth in the presence of 100 μM H_2_O_2_ in the cell culture medium was also here assessed. After 24 h of cell growth in the presence of 100 μM H_2_O_2_, we observed a decrease of about 68% of the cell viability in the presence of neat PFM (as control), while only about a 27% of decrease occurred in the presence of PFM+GaOS (Figure 5c). Therefore, the cellular protection from oxidative stress was a peculiarity of PFM+GaOS, which was not obtained in the presence of PFM + Na_2_S, where a decrease of about 78% of cell viability, comparable to the control (PFM), was observed (Figure 5c). These results were in agreement with the property of the garlic OSCs to perform a prolonged and gradual H_2_S release over time, which was not obtainable using Na_2_S. The prolonged H_2_S slow-release most likely leads to an antioxidant protective effect on cells, thereby stimulating cell growth. However, it was not possible to completely exclude that the effect was also due to the formation of other gaseous species able to reduce the oxidative action of the hydrogen peroxide. A previous study on H_2_S-releasing poly(ε-caprolactone) (PCL) fibers [25] demonstrated that the H_2_S-release induced an anti-proliferative and synergistic effect with H_2_O_2_ on H9c2 cardiomyoblasts in the absence of cysteine in the cell culture medium. By contrast, in our case a stimulating effect of the PFM+GaOS on the cell proliferation was observed also in the absence of cysteine, probably due to several factors, including the intrinsic properties of the used H_2_S-releasing agent and the ability of PLA fibers to generate a slow H_2_S-release. Although, the H_2_S-release/hydrogen peroxide/oxidative stress system is extremely complex and the in vitro results are not easily translatable to an in vivo setting, our results on the cyto-protective activity of H_2_S-releasing fibrous mats may be also in agreement with recent studies on different H_2_S-releasing fibers [26,47] that were able to promote wound healing in vivo through cyto-protection. This property was likely due to the ability of the H_2_S to reduce the inflammatory response and the oxidative damage, and to stimulate the angiogenesis [49]. Therefore, H_2_S-releasing mats, like the H_2_S-releasing molecules [48,50,51], may promptly scavenge hydrogen peroxide, increasing pro-cell survival signaling and, at the same time, decreasing pro-apoptotic signaling. The protective effect of the H_2_S-releasing PFM from oxidative damage, which was observed in the non-direct growth on the membranes, opens new perspectives in their application as biomedical devices that could be used inside or outside the body, such as non-implantable devices/patches for wound dressing, implantable material like vascular grafts, heart valves and implants for reducing the ischemic damages and improving the patient health and medical conditions. Moreover, the enhancement of the wound healing induced by *Allium sativum* L. via re-epithelialization and neovascularization has been already demonstrated [52,53].

### 2.4. Microstructural and Mechanical Properties of GaOS and DADS Functionalized PFM

**Figure 6:**
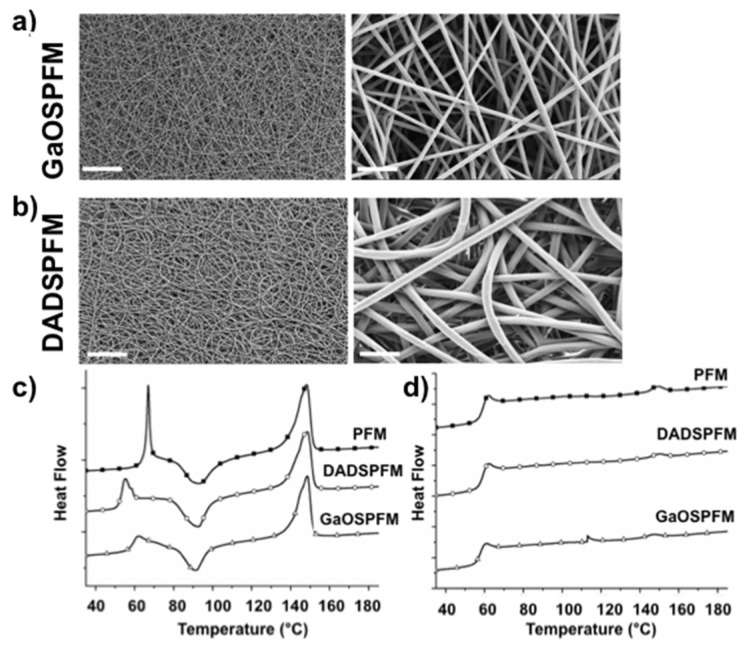
Microstructural and thermal characterization of functionalized PFM. SEM micrographs of (**a**) GaOSPFM and (**b**) DADSPFM (**left**: magnification 1k×, scale bar 50 µm, **right**: magnification 10k×, scale bar 5 µm); Differential scanning calorimetry (DSC) curves related to the first (**c**) and second (**d**) heating scans of PFM, DADSPFM and GaOSPFM.

The synthesis of functionalized PFM with GaOS or DADS, which is a component of the GaOS, could represent an improving in the production of the H_2_S-releasing PFM ready for use and the release of the signaling molecule more stable over time. Therefore, an *ex-novo* synthesis of functionalized PFM with the encapsulation of GaOS or DADS, as pure garlic OSC, was here performed. In Figure 6, SEM micrographs of the functionalized PFM are compared. In all cases, defect-free randomly oriented fibers were obtained with a uniform distribution of the OSCs along the fibers, and crystals were not detected within the fibers and on their surface. The presence of DADS led to bigger fibers (DADSPFM, Figure 6b), with an average diameter of 1.21 ± 0.2 µm (Table 1), than both PFM (average diameter of 0.71 ± 0.2 µm, Table 1, Figure 2a) and GaOSPFM (average diameter of 0.65 ± 0.10 µm) (Table 1, Figure 6a). Moreover, the DADS addition favoured the deposition of more rounded fibers (Figure 6b), whereas the GaOS functionalization did not alter the fiber morphology (Figure 6a), resulting very similar to PFM (Figure 2a).

These results were in agreement with previous studies on the production of PCL fibers loaded with the H_2_S donor *N*-(benzoylthio)benzamide (NSHD1) [25,26]. However, in that case the NSHD1 addition to PCL solutions during the synthesis did not influence the morphology and the average diameter of the PCL fibers [25,26]. On the contrary, we evidenced significant differences between PFM and DADSPFM, as described above, suggesting an interaction between DADS and PLA. Furthermore, DSC analyses and uniaxial tensile tests were performed on the PFM, in order to investigate the influence of GaOS and DADS on the thermal and mechanical properties of the produced fibers, respectively. Indeed, it is well known that the scaffold topographical, chemico-physical, and mechanical properties can play a pivotal role in the stem cell differentiation towards specific phenotypes as well as on the preservation of their stem cell status [54,55], even if the underneath mechanisms of this phenomenon (mechanotransduction) have not been fully comprehended and identified. The glass transition (T_g_), melting (T_m_), cold crystallisation (T_cc_) temperatures, the melting (ΔH_m_) and cold crystallization (ΔH_cc_) enthalpies, and the crystallinity degree (χ), related to the first and the second heating scans, are summarised in Table 2.

The first and second heating scan DSC curves are compared in Figure 6c,d, respectively. By comparison of the first heating scans data in Table 2, the OSCs addition did not significantly influence the melting temperature (T_mI_). Indeed, in all cases, it was possible to detect two melting peaks in the first heating scan DSC curves: a first very broad little shoulder (T_m1I_), followed by a well-defined melting temperature (T_m2I_), due to the coexistence of two PLA crystalline structures [56,57] or due to the dual lamellae population [57]. In details, the lower melting shoulder is ascribed to the crystals formed through a melt-recrystallization process during the heating scan (cold crystallization process), whereas the highest well defined one is associated to the melting of the original crystals derived from the sample preparation [56,57].

On the contrary, OSCs addition led to a significant decrease of T_g_ value, particularly in the case of DADSPFM, suggesting an interaction between the OSCs and the PLA chains, and a plasticizing effect of the OSCs.

**Figure 7:**
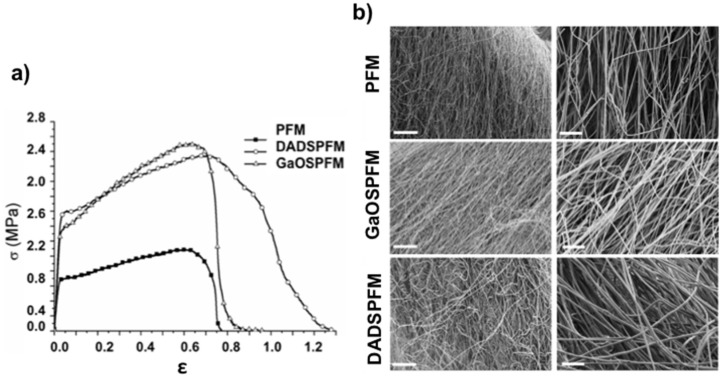
Mechanical properties of functionalized PFM. (**a**) Stress-strain curves of PFM, GaOSPFM, and DADSPFM; (**b**) SEM micrographs of the related fracture stress-strained surfaces (**left**: magnification 1k×, scale bar 50 µm, **right**: magnification 5k×, scale bar 10 µm).

Moreover, plasticizers are able to increase polymer chain mobility and flexibility by decreasing intermolecular forces [58] and hydrogen bonding between polymer chains [59,60]. In this manner, the plasticizers presence also tends to promote the cold crystallization process, as testified by the slightly lower T_cc_ values detected for GaOSPFM (91 °C) and DADSPFM (92 °C) with respect to PFM one (94 °C). Indeed, the exothermic peak at around 91–94 °C was ascribed to the PLA typical cold crystallization (T_cc_), characterised by the reorganization of amorphous domains into crystalline regions, promoted by the enhanced macromolecular flexibility and mobility upon increasing temperature. Thus, the promoted crystallization led to higher crystallinity degree values in the case of OSCs functionalised fibers (Table 2), particularly for GaOSPFM (11.4% for GaOSPFM vs. 7.8% for PFM). In all cases, the electrospun mats are generally characterised by very low crystallinity degree values, since the electrospinning process favours a rapid evaporation of the solvent and, thus, a fast solidification of stretched chains during the later stages of the process, due to the high elongation strain rate of the electrospinning jet. Consequently, the stretched chains do not have enough time to reorganize themselves before the solidification [61] and the majority of the chains are in the amorphous state. As far as the second heating scan data were concerned, interestingly the glass transition values were very comparable, the cold crystallization phenomenon was not revealed, and the melting was hardly detectable, indicating that, in these samples, the applied cooling conditions were not able to induce the complete PLA crystallization. Moreover, as expected, significantly lower crystallinity degree values were obtained in all cases, due to the elimination of the thermal history, particularly of the influence of the applied process for the production of the fibers and of the fibrous structure, in agreement with previous studies [29,62,63]. Furthermore, the influence of the added OSCs on the mechanical properties of the obtained fibers was studied by means of tensile tests. The stress-strain curves of neat and functionalised PFM are compared in Figure 7a and the data of the uniaxial tensile tests, in terms of ultimate tensile stress (σ_max_), yield stress (σ_s_), and Young’s modulus (E), are reported in Table 1.

A remarkably significant increment of the mechanical properties, in terms of Young modulus (E) and σ_max_ (Table 1), was recorded in the case of functionalized PFM with respect to neat PFM (E = 28 ± 1 MPa and σ_max_ = 1.1 ± 0.1 MPa): E = 65 ± 18 MPa and σ_max_ = 2.4 ± 0.2 MPa for DADSPFM; E = 52 ± 6 MPa and σ_max_ = 2.7 ± 0.3 MPa for GaOSPFM. It has to be taken into account that the mechanical properties of electrospun mats are strongly influenced by several factors that interact each other: (i) intrinsic characteristics of the polymer which influence the fibers deformation mechanisms (whose increment causes a higher ultimate tensile stress); (ii) fibers packing density, interaction, and mechanical interlocking that increment the elastic modulus; (iii) fibers average diameter size (thinner fibers correspond to a higher packing density); and (iv) possible presence of defects (e.g., beads) in the fibers [28,64].

Moreover, by comparing the SEM micrographs of the stress-strained functionalized PFM surfaces (Figure 7b), the neat PFM and GaOSPFM presented comparable behaviour, with tendency of the fibers to align in the load direction. On the contrary, DADSPFM showed lower fiber alignment degree, due to its different morphological features (Figure 6) with respect to PFM and GaOSPFM. Thus, it is possible to conclude that among the several factors able to simultaneously affect the mechanical behaviour of fibrous mats, the predominant one seems to be the different chemical composition, suggesting an interaction between the sulphur compounds and the polymeric chains, in agreement with DSC data, that evidenced an increased crystallinity degree in the case of functionalized PFM (Table 2). Indeed, improved mechanical properties were revealed for both OSCs functionalized fibers, even if GaOSPFM resulted morphologically comparable to neat PFM (Figure 2a and Figure 6a), presenting comparable average diameter size (Table 1) and, thus, the same interweave degree. Therefore, it is possible to properly modulate the chemico-physical properties of the produced fibrous mats by the addition of OSCs in order to monitor their biological responsiveness.

### 2.5. Biological Properties of GaOS and DADS Functionalized PFM

**Figure 8:**
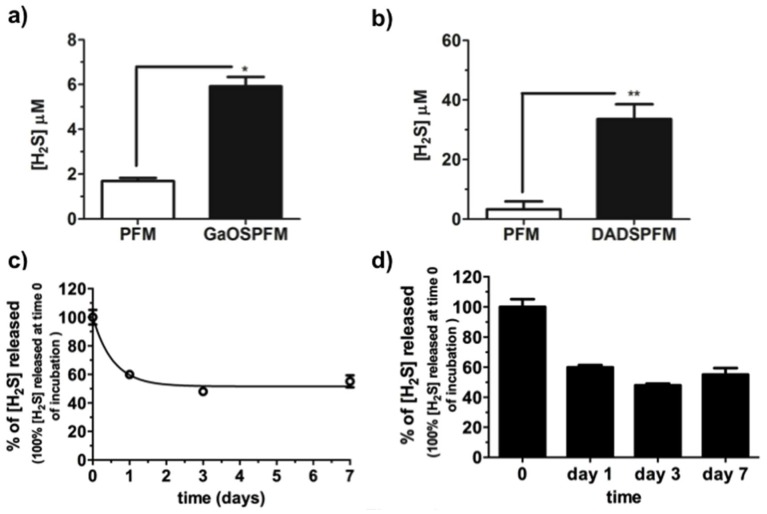
H_2_S release from functionalized PFM. H_2_S release from (**a**) GaOSPFM and (**b**) DADSPFM disks (1 cm of diameter) performed with 1 h of incubation at 37 °C; H_2_S release from DADSPFM disks over time, after incubation: (**c**) in buffer solution (50 mM Tris HCl, pH 8.0) at 37 °C, and (**d**) dried in petri dish at room temperature for 0, 1, 3, and 7 days. * p < 0.05; ** p < 0.02.

The H_2_S-releasing properties of GaOSPFM and DADSPFM were assessed and both PFM were able to release H_2_S (Figure 8a,b). However, the H_2_S-release was very low and less than that observed in the case of PFM doped using 10 μL of GaOS. In particular, the release from GaOSPFM was less than that from DADSPFM.

This result indicated that the concentration of the H_2_S-donors was very low in the GaOSPFM, probably due to chemical reactions between the OSCs and the used solvents, which occurred during the PFM synthesis. By contrast, DADSPFM maintained the property to release H_2_S even after several days from the synthesis of the fibers (Figure 8b–d): after 7 days of incubation in buffer solution the membranes were still able to release 55.2 ± 4.2% (Figure 8c) of the H_2_S produced without incubation, while the 66.4 ± 6.7% was obtained in dried conditions. In this last case, further, during the first three days, no significant changes in the H_2_S release from DADSPFM were observed (Figure 8d).

These data show that the optimized DADSPFM synthesis is able to preserve the DADS properties and to guarantee its controlled release.

**Figure 9:**
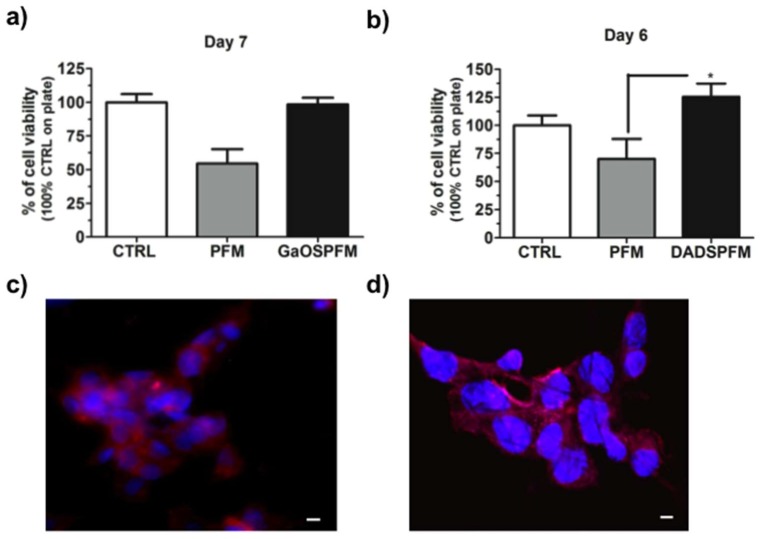
Biological properties of functionalized PFM. Cell viability of cMSC cultured on functionalized PFM: (**a**) on GaOSPFM and (**b**) on DADSPFM after 7 and 6 days of growth, respectively; (**c**) fluorescence micrograph of cMSC seeded on DADSPFM after 6 days and (**d**) confocal micrograph of cMSC seeded on DADSPFM after 7 days of culture. The nuclei are stained with Hoechst 33342 (in blue) and the phalloidin is in red. Scale bars = 10 µm. * p < 0.05.

In order to assess the potential application of the produced systems as platforms for tissue regeneration, GaOSPFM and DADSPFM were used as scaffolds for 2D-cultures of cMSC (Figure 9). Both the produced PFM did not show a cytotoxic effect and, in particular, the DADSPFM were also able to stimulate the cMSC growth (Figure 9b).

The fluorescence micrographs (Figure 9c,d) showed a good adhesion of the cMSC to the DADSPFM and cell-cell interaction after 6 and 7 days of cell culture. These results were also in agreement with our previous studies [24,34], where the hydrogel scaffolds and DADS based nanoemulsions were able to increase the cMSC proliferation and α-sma expression. Therefore, H_2_S-relasing fibrous scaffolds might stimulate the tissue repair by activating adult resident stem cells. Moreover, such as previously observed for H_2_S-donor molecules [65,66], the DADSPFM could activate the Akt signaling pathways and, thus, could be considered for promoting the endothelial cell growth, migration, wound healing features, capillary morphogenesis, and neovascularization of implants.

## 3. Materials and Methods

### 3.1. Extraction of GaOS from Allium sativum L.

GaOS was prepared as previously described by Buyhan et al. (2015) [30]. Briefly, 5 g of garlic cloves were cut in 50 mM Tris-HCl buffer pH 7.5 at room temperature for about 5–10 min and then the crushing was carried out in liquid N_2_ and centrifuged. The precipitated fraction was incubated in absolute EtOH for 24 h at room temperature and after centrifugation at 13,439× *g* the ethanol soluble fraction (GaOS) was stored at −20 °C. RP-HPLC analysis was performed using mod. LC-10AVP (Shimadzu, Milan, Italy), equipped with a C_18_ column (150 mm × 4.6 mm, 5 μm, CPS Analitica, Rome, Italy), using 0.1% trifluoracetic acid as solvent A and 80% CH_3_CN, 0.1% trifluoracetic acid as solvent B and with a solvent B gradient (0–5 min, 0%; 5–55 min, 60%; 55–60 min, 60% and 65–85 min 90%). Elute was monitored at 220 nm by UV detector (Shimadzu, Milan, Italy).

### 3.2. PFM Synthesis and Characterization

#### 3.2.1. Fabrication of Doped PFM

Neat PFM were produced by electrospinning. A proper amount of PLA pellets (3051D, Nature Works, Minnetonka, MN, USA, specific gravity of 1.25 g/cm^3^, molecular weight (M_n_) of ca. 1.42 × 10^4^ g/mol) (15 % *w*/*v* with respect to the used solvents) was dissolved in CHCl_3_:DMF (67:33, in vol. ratio), under continuous magnetic stirring. The obtained solution was poured in a glass syringe (Socorex, Ecublens, Switzerland), equipped with a 18 G needle, fixed in a digitally controlled syringe pump (KD Scientific, Holliston, MA, USA), and electrospun in air at room temperature, setting an applied voltage of 12 kV, a flow rate of 0.5 mL/h and a needle-target distance of 15 cm. The resultant mats were dried under vacuum for 24 h and stored in a desiccator.

Disks (0.5 or 1 cm of diameter) of the produced PFM were cut, sterilized using ethanol and by exposition at UV, and after were doped with GaOS extract by drop casting and dried.

#### 3.2.2. Fabrication of Functionalized PFM

OSCs functionalized PFM were prepared by electrospinning technique, using either the GaOS extract or DADS (Sigma Aldrich, Milan, Italy). OSCs (5% *v*/*v* with respect to the used solvents) and PLA (15 % *w*/*v* with respect to the used solvents) based solutions were prepared by dissolving proper amounts of PLA pellets and OSCs in CHCl_3_: DMF (67:33 *v*/*v*). Prepared solutions were poured in a glass syringe, equipped with an 18 G needle, fixed in a digitally controlled syringe pump, and electrospun in air at room temperature, setting an applied voltage of 12 kV, a flow rate of 0.5 mL/h and a needle-target distance of 15 cm.

#### 3.2.3. Characterization of PFM

The morphology of the PFM was investigated by means of field emission gun scanning electron microscopy (FEG-SEM, Leo Supra 35, Cambridge, UK), equipped with the energy dispersive X-ray spectroscopy (EDS, INCA Energy 300, Oxford ELXII detector, Abingdon, UK). The average fiber diameter was calculated by means of ImageJ (NIH). The thermal properties were measured by differential scanning calorimetry (DSC, TA Instruments Q2000) in the following conditions: sample weight ~5 mg, nitrogen flux 50 cc/min, range temperature 0–250 °C, heating and cooling rates 10 °C/min. Melting temperatures (T_mI_ and T_imid_), cold crystallization temperatures (T_ccI_ and T_ccII_), melting enthalpies (ΔH_mI_ and ΔH_mII_), cold crystallization enthalpies (ΔH_ccI_ and ΔH_ccII_), and crystallinity degrees (χ_I_ and χ_II_) were evaluated in the first and in the second heating scans.

The crystallinity degree (χ) was calculated as:

(1) $$\;\chi =\frac{{\Delta H}_{m}-{\Delta H}_{\mathrm{cc}}}{{\Delta H}_{0,m}}\times 100\;$$

where ΔH_m_, ΔH_0,m_, and ΔH_cc_ represent the acquired melting enthalpy, the melting enthalpy for 100% crystalline PLA material (i.e., 93 J/g) [67] and the acquired cold crystallization enthalpy, respectively.

Mechanical properties of PFM were investigated by uniaxial tensile tests performed on dog-bone specimens (width 4.8 mm, length 22.25 mm), at 1.2 mm/min to rupture by an electromechanical machine (Lloyd LRX, Fareham, UK), equipped with a 50 N load cell, following ASTM D882 standard. Four specimens were prepared for each electrospun matrix, at least. All mechanical properties were calculated considering the nominal specimen cross-section. Finally, the fracture surface of stress-strained PFM was investigated by means of SEM.

### 3.3. H_2_S Releasing Assay

The H_2_S release from GaOS extract, GaOS doped, and OSCs functionalized PFM was estimated by methylene blue (MB) assay, which consists in the reaction between sulfide and *N*,*N*-dimethyl-*p*-phenylenediaminesulphate, in the presence of the oxidizing agent Fe^3+^ in hydrochloric acid, to form methylene blue, involving a 1:2 stoichiometry [30]. Briefly, 25 μL of GaOS (42 mg d.w./mL) solution or PFM based disks (diameter of 1 cm) were incubated in 150 μL of 50 mM Tris HCl, 8.0 pH, buffer and 1 mM of dithiothreitol (DTT) at 37 °C for 30 min (in case of the solutions) or 60 min (in case of the PFM). The MB was produced by addition of 20 μL of solution I (20 mM *N*′,*N*′-dimethyl-*p*-phenylenediaminedihydrochloride in 7.2 M HCl) and 20 μL of solution II (30 mM FeCl_3_ in 1.2 M HCl) and mixing for 10 min at room temperature. The concentration of MB in the supernatants was then spectrophotometrically assessed at 670 nm. The results were plotted using GraphPad Prism version 5.0 for Windows (GraphPad Software, San Diego, CA, USA). The H_2_S concentrations were calculated using a calibration curve obtained at different concentrations of Na_2_S. Each bar represents the ±SD of three experiments as biological replicas (Figure S1).

### 3.4. Antimicrobial Activity

The antimicrobial activity was tested using ampicillin resistant *E. coli* (*E. coli*^AmpR^) BL21 strain grown onto petri dishes with agar-LB medium with 100 µg/mL of ampicillin. The disks (diameter of 1 cm) were previously sterilized using ethanol and UV exposure and then were doped with 10 or 25 μL of GaOS extract; the control was performed using 25 μL of ethanol without GaOS. After the evaporation of the solvent, the disks were placed on the petri dishes seeded with the bacteria and incubated at 37 °C overnight. The inhibition of the bacterial growth around the disks was analysed.

### 3.5. Stem Cell Proliferation on PFM

Cell studies were performed using human Lin^−^ Sca-1^pos^ cardiac mesenchymal stem cells (cMSC) line, which was obtained and characterized as previously described [68,69], using cells isolated from human auricular biopsies made during the course of coronary artery bypass surgery of patients (seven male, six female, 52–83 years) undergoing cardiac surgery who accepted to be enrolled in the research study after signing a written consent form according to a joint protocol approved by the Ethic Committees of Ospedale Maggiore della Carità, Novara and University Hospital Le Molinette, Turin 2011.

Cell cultures were performed in Dulbecco’s Modified Eagle Medium (DMEM) with high glucose (Gibco, Italy), containing 10% Fetal Bovine Serum (FBS) (Gibco, Monza, Italy), 1% penicillin-streptomycin, 1% l-Glutamine-Penicillin-Streptomycin solution (Sigma-Aldrich, Italy).

The biological responsiveness of the produced systems was investigated by in vitro cell viability of cMSC, up to 7 days, using either the methylthiazolyldiphenyl-tetrazolium bromide (MTT) assay [70] or the Water-Soluble Tetrazolium salt (WST-1) assay (4-[3-(4-lodophenyl)-2-(4-nitrophenyl)-2H-5-tetrazolio]-1,3-benzene disulfonate) (Roche Diagnostics, Sigma Aldrich, Italy) [71].

### 3.6. Immunofluorescence Analysis

To assess the stem cells phenotype, cMSC were seeded on the PFM scaffolds cultured for 7 days in complete medium. Scaffolds were then washed in PBS, fixed in 4% paraformaldehyde (PFA) in PBS for 15 min at room temperature and permeabilized with 0.2% *v/v* Triton X-100 (Sigma-Aldrich, Italy) for 10 min. Then they were incubated with antibodies specific for α-smooth muscle actin (α-sma) (Sigma-Aldrich, Italy), followed by the appropriate 488-Alexa fluorochrome-conjugated secondary antibodies (ThermoFisher Scientific, Rodano, Italy), or incubated with 488-Alexa fluorochrome-conjugated phalloidin (Life Science, Rodano, Italy). Nuclei were stained with Hoechst 33342 (Sigma-Aldrich, Italy). Confocal microscopy of the cell-seeded constructs was performed using a LSM 700 Confocal Laser Scanning Microscope (Carl Zeiss MicroImaging, Jena, Germany) and acquired by means of ZEN 2010 software.

### 3.7. Statistical Analysis

The molecular analysis and cell survival were expressed as mean ± standard deviation (SD). Stem Cell survival was analysed using One-way ANOVA test or the Student’s *t*-test. *p* Values < 0.05 were considered significant.

## 4. Conclusions

New H_2_S slow-releasing PFM were here manufactured and characterized for their morphological, thermal, mechanical, and biological properties. Two different procedures were used for the production of PFM able to provide H_2_S slow-release: (i) the direct doping of PFM with natural garlic OSCs by drop casting GaOS on the surface of neat fibrous mats and (ii) OSCs direct encapsulation within PFM, using both GaOS and DADS. The produced H_2_S-releasing PFM were homogeneous and defect-free with significant increment of the mechanical properties. We show for the first time the capability of the antimicrobial H_2_S-releasing fibers to stimulate the cMSC proliferation, as well as their ability to reduce the oxidative damage induced by hydrogen peroxide. On this basis, a potential and future application for the PFM+GaOS and DADSPFM could also be for the wound-dressing sector. Moreover, the data here presented suggested the possible exploitation of these low-cost H_2_S-releasing fibers as antimicrobial fibrous scaffolds/patches, able to improve the stem cell proliferation and stimulate the tissue regeneration.

## Figures and Tables

**Table 1:** Average diameter values and mechanical properties of the produced neat and functionalized PFM.

Sample	Average Diameter (µm)	E (MPa)	σ_max_ (MPa)	σ_y_ (MPa)	ԑ_max_
PFM	0.71 ± 0.2	28 ± 1.0	1.1 ± 0.1	0.63 ± 0.01	1.0 ± 0.2
GaOSPFM	0.65 ± 0.1	52 ± 6.0	2.7 ± 0.3	1.5 ± 0.1	1.0± 0.2
DADSPFM	1.21 ± 0.2	65 ± 18	2.4 ± 0.2	1.6 ± 0.3	1.3 ± 0.1

**Table 2:** Differential scanning calorimetry (DSC) data for PFM, GaOSPFM and DADSPFM.

**Sample**	**I Heating**						
	**T_gI_ (°C)**	**T_ccI_ (°C)**	**ΔH_ccI_ (J/g)**	**T_m1I_ (°C)**	**T_m2I_ (°C)**	**ΔH_mI_ (J/g)**	**χ_I_ (%)**
PFM	65.9	94.0	19.2	145.9	148.8	26.5	7.8
GaOSPFM	59.6	91.5	14.9	145.4	148.4	25.5	11.4
DADSPFM	53.5	92.7	18.2	146.3	148.6	26.1	8.5
	**II Heating**						
	**T_gII_ (°C)**	**T_ccII_ (°C)**	**ΔH_ccII_ (J/g)**	**T_m1I_ (°C)**	**T_m2II_ (°C)**	**ΔH_mII_ (J/g)**	**χ_II_ (%)**
PFM	59.2	-	-	-	149.8	1.1	1.2
GaOSPFM	58.1	-	-	-	146.8	0.8	0.8
DADSPFM	59.0	-	-	-	149.7	0.7	0.8

## Supplementary Materials

Supplementary materials can be found at http://www.mdpi.com/1422-0067/19/8/2368/s1.

## Abbreviations

ARE	antioxidant response element
cMSC	human Lin^−^ Sca1^+^ cardiac mesenchymal stem cells
DADS	diallyldisulfide
DADSPFM	poly(lactic) acid fibrous membranes functionalized with DADS
DMF	dimethylformamide
DSC	differential scanning calorimetry
DTT	dithiothreitol
d.w.	dried weight
ECM	extracellular matrix
EDS	energy dispersive X-ray spectroscopy
FBS	Fetal Bovine Serum
FDA	Food and Drug Administration
FEG-SEM	field emission gun scanning electron microscopy
GaOS	garlic oil-soluble extract
GaOSPFM	poly(lactic) acid fibrous membranes functionalized with GaOS
GSH	glutathione
MB	methylene blue
Nrf2	Nuclear factor (erythroid-derived 2)-like 2
NSHD1	*N*-(benzoylthio)benzamide
PCL	Polycaprolactone
PFM	poly(lactic) acid fibrous membranes
PLA	poly(lactic) acid
TCP	tissue culture on plate
T_m_	melting temperature
α-sma	α-smooth muscle actin
ΔH_m_	melting enthalpy
ΔH_cc_	crystallization enthalpy
χ	crystallinity degree
