# Supplementary material for: Hydrogen Sulfide-Releasing Fibrous Membranes: Potential Patches for Stimulating Human Stem Cells Proliferation and Viability under Oxidative Stress
Source: Int J Mol Sci. 2018 Aug 11;19(8):2368. doi: 10.3390/ijms19082368 (PMC6121677; doi:10.3390/ijms19082368)
Supplement: Supplementary file 1 [file ijms-19-02368-s001.pdf]

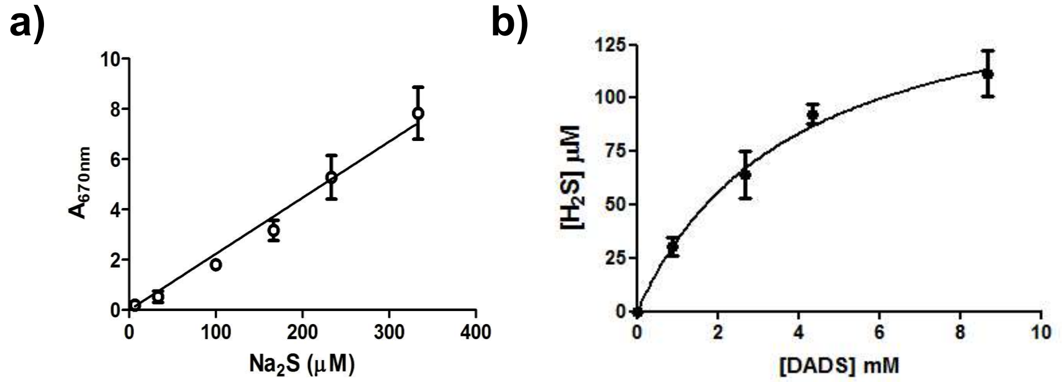

Figure S1

Figure S1. H_2S slow-releasing from Na_2S and DADS. **a**) Calibration curve of H_2S formation by methylene blue assay, obtained at different concentration of Na_2S by absorbance 670 nm. **b**) H_2S-release at different concentrations of DADS in DMSO, in the presence of 1 mM DTT in 50 mM Tris-HCl, pH 7.4 buffer. The H_2S concentrations were calculated using a calibration curve obtained at different concentrations of Na_2S. Each bar represents the ±SD of three experiments as biological replicas.

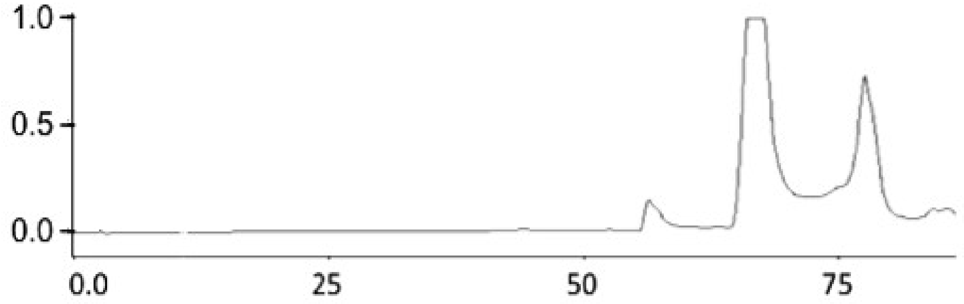

Figure S2

Figure S2. RP-HPLC of DADS. RP-HPLC chromatogram of DADS obtained using C_{18} column at 0.8 ml/min flow rate. The elution was performed with a linear gradient of solv. B (80%CH_3CN, 0.1% TFA).

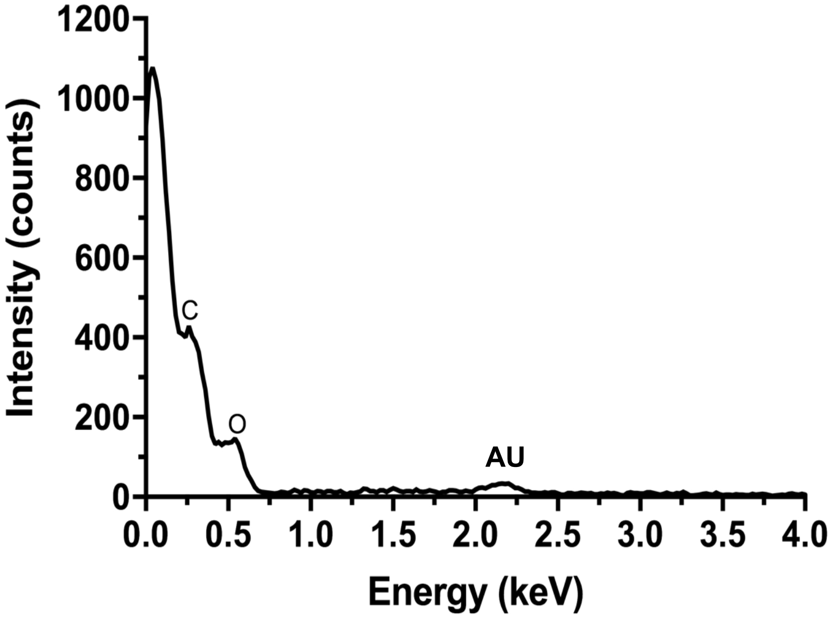

Figure S3

Figure S3. EDS of neat PFM

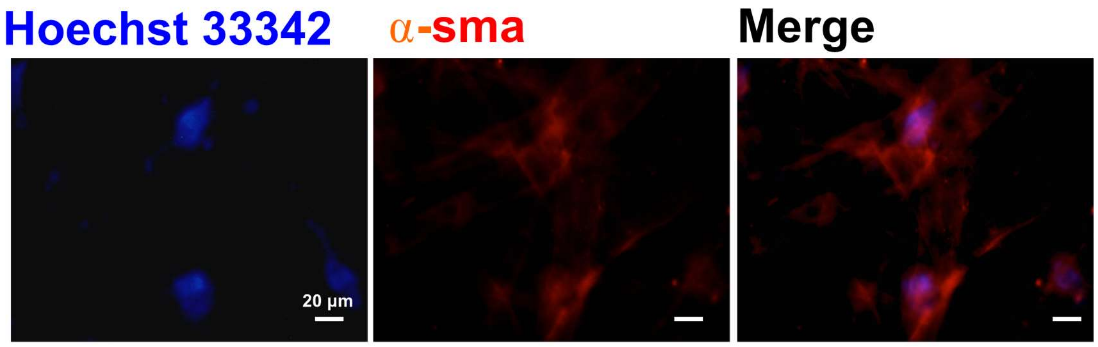

Figure S4

Figure S4. Fluorescence confocal micrographs of cMSC cultured on PFM for 3 days. The nuclei are stained with Hoeschst 33342 (in blue) and the expressions of α-sma (in red) are detected. Scale bars= 20 μm.
